# *Tomato yellow leaf curl virus* infection mitigates the heat stress response of plants grown at high temperatures

**DOI:** 10.1038/srep19715

**Published:** 2016-01-21

**Authors:** Anfoka Ghandi, Moshe Adi, Fridman Lilia, Amrani Linoy, Rotem Or, Kolot Mikhail, Zeidan Mouhammad, Czosnek Henryk, Gorovits Rena

**Affiliations:** 1 Faculty of Agricultural Technology, Department of Biotechnology, Al-Balqa’ Applied University, Al-Salt 19117, Jordan; 2 Institute of Plant Sciences and Genetics in Agriculture, Robert H. Smith Faculty of Agriculture, Food and Environment, Hebrew University of Jerusalem, Rehovot 76100, Israel; 3 Department of Plant Pathology and Microbiology, The R.H. Smith Faculty of Agriculture, Food and Environment, The Hebrew University of Jerusalem, Rehovot 76100, Israel; 4 Department of Biochemistry, Tel-Aviv University, Ramat Aviv 69978, Israel; 5 Al-Qasemi Research Center (QRC), Molecular Genetics and Virology, Baqa-El-Gharbia 30100, Israel

## Abstract

Cultured tomatoes are often exposed to a combination of extreme heat and infection with *Tomato yellow leaf curl virus* (TYLCV). This stress combination leads to intense disease symptoms and yield losses. The response of TYLCV-susceptible and resistant tomatoes to heat stress together with viral infection was compared. The plant heat-stress response was undermined in TYLCV infected plants. The decline correlated with the down-regulation of heat shock transcription factors (HSFs) *HSFA2* and *HSFB1*, and consequently, of HSF-regulated genes *Hsp17*, *Apx1, Apx2* and *Hsp90*. We proposed that the weakened heat stress response was due to the decreased capacity of HSFA2 to translocate into the nuclei of infected cells. All the six TYLCV proteins were able to interact with tomato HSFA2 *in vitro*, moreover, coat protein developed complexes with HSFA2 in nuclei. Capturing of HSFA2 by viral proteins could suppress the transcriptional activation of heat stress response genes. Application of both heat and TYLCV stresses was accompanied by the development of intracellular large protein aggregates containing TYLCV proteins and DNA. The maintenance of cellular chaperones in the aggregated state, even after recovery from heat stress, prevents the circulation of free soluble chaperones, causing an additional decrease in stress response efficiency.

In the Middle East and in other tropical and sub-tropical countries, tomatoes grown in the field in the spring and summer, are exposed to high temperatures (40 ^o^C and higher), often in combination with *Tomato yellow leaf curl* virus (TYLCV) infection. TYLCV englobes a number of viruses (family *Geminiviridae*, genus *Begomovirus*) infecting tomato cultures (*Solanum lycopersicum*) worldwide^1^. Following the inoculation of virions in the plant vascular system by the whitefly *Bemisia tabaci*, the virus reaches the phloem-associated cells. The viral ssDNA genome is shuttled by the coat protein into the nucleus where it replicates *via* a dsDNA intermediate encoding into six multifunctional proteins. Newly synthesized coat protein (CP) and viral genomes are assembled into viral particles, which move to the cytoplasm and translocate to neighboring cells and long-distance via the vascular system, from where they are acquired by the whitefly vector during feeding^2^. Geminiviruses reprogram the cell cycle of mature plant cells, interacting with host factors to create a permissive environment for viral replication^3^. TYLCV CP recruits HSP70, one of the main cellular chaperones, to promote CP translocation into the nuclei of infected cells^4^.

To ensure a successful long-term infection cycle, geminiviruses must restrain their destructive effect on the host cells and prevent drastic plant responses. Recently, we described the suppression of host cell death, induced by inhibition of HSP90 and its co-chaperone SGT1, in tomato plants infected by TYLCV^5^. The accumulation of damaged ubiquitinated proteins and inhibition of 26S proteasome were significantly relieved under TYLCV infection. Moreover, HSP90-dependent activation of the heat shock transcription factors (HSFs: HsfA2 and HsfB1) and of the downstream genes *Hsp17* and *Apx1/2* (ascorbate peroxidase) under heat stress (HS) conditions were suppressed in TYLCV-infected tomatoes. Following suppression of the plant stress response, TYLCV can replicate and accumulate in a permissive environment.

One of the broadly used host protection mechanisms in preventing viral replication and spread involves the destruction of viral components, frequently CP, by cellular proteolytic degradation systems such as the 26S proteasome and autophagy. The degradation of TYLCV CP was recently described^6^. TYLCV counters proteolysis by inducing the aggregation of viral proteins such as the CP^7^ and V2 proteins^8^, thereby limiting the accessibility of the viral proteins to cellular degradation. Indeed, the progress of TYLCV infection was shown to be accompanied with the development of CP aggregates of increasing size, first in the cytoplasm and then in the nucleus of infected tomato cells^7^. Large nuclear aggregates contain infectious virus particles that are transmissible to test tomato plants by *B. tabaci*. In contrast to TYLCV-susceptible (S) tomatoes, TYLCV-resistant (R) plants respond to TYLCV by a significant delay in the formation of large aggregates^7^. Accordingly, we proposed that the accumulation of large TYLCV aggregates is an indicator of a successful viral infection.

Elevated temperatures interfere with plant-pathogen interactions, often compromising R gene-mediated disease responses, including the hypersensitive response (HR)^9^. At high temperatures, the collapse of the *Nicotiana tabacum* N gene-mediated *Tobacco mosaic virus* (TMV) resistance was caused by heat-induced conformational changes of the plant R protein and was associated with downregulation of NADPH oxidase and superoxide and stimulation of dehydroascorbate reductase^10^. Mild increases in temperature also compromised the R gene-mediated HR following expression in *N. benthamiana* of *Potato virus X* (PVX) CP or of TMV helicase^11^. At high temperatures, *Tomato spotted wilt virus* (TSWV) suppressed the TSWV-mediated HR in pepper plants (*Capsicum annuum*)^12^. The combination of HS, drought and *Turnip mosaic virus* (TuMV) infection was investigated in *Arabidopsis*^13^. A significant reduction in biomass was found in all single stress conditions, which was further exaggerated when the different stresses were applied together, especially, under a combination of virus and heat stresses. Moreover, heat or heat and drought increased the susceptibility of *Arabidopsis* plants to virus infections. The cause for this increased susceptibility was claimed to reside in an altered expression of components of the signal transduction pathway and/or in a modified metabolite signaling.

Increasing temperatures involve reprogramming of signal transduction components, transcription factors and proteins associated with the metabolism of stress-generated reactive oxygen species (ROS) (reviewed in^14^ and references therein). Transcript profiling of tomato plants showed that genes affected by high temperatures included those encoding for heat shock proteins (HSPs), osmolytes, enzymes that affect the membrane fluidity and enzymes involved in ROS homeostasis^15^. Further analysis suggests that high temperature response requires a coalition of pathways that culminate in the activation/synthesis of HSFs and accumulation of HSPs. Plants possess a larger number of *Hsf* genes than animals, leading to the hypothesis that HSFs have gained additional functions in plants^16^. Support for this hypothesis comes from the overexpression of HSFA2 in transgenic Arabidopsis, which resulted in an increased tolerance to combined light and HS^17^. In addition, different biotic stresses induce HSF expression indicating that they may also play a role in pathogen defense^16^. Since biotic- and abiotic stress-dependent signaling pathways may act antagonistically^18^, the control of these pathways is essential for an effective plant response.

In the current study, we examined whether the tomato defenses against TYLCV infection were influenced by HS or whether the virus affected HS response. In general, plant immunity occurs at different levels and can be divided into basal and R gene-mediated resistance^19^. However, we have shown before that TYLCV infection does not induce R gene-mediated resistance^20^. Furthermore, only some key markers of basal resistance, such as pathogen related proteins (PR), were up-regulated by TYLCV, and only at the late stages of infection^21^,^22^. These data allowed us to omit here the analysis of R genes and PR genes patterns in our experiments and to emphasis the changes in the patterns of several key HSPs, HSFs and HSF-regulated genes. In tomato cell cultures subjected to heat stress, the constitutively expressed HS transcription factor HsfA1 is complemented by two HS-inducible forms, HsfA2 and HsfB1. Because of its stability, HsfA2 accumulates to high levels during prolonged HS and recovery. It tightly regulates the expression of the APX gene family, which may play a major role in removing intercellular H_2_O_2_ and preventing ROS overproduction^23^. Therefore, we have initiated the investigation of the combined effect on tomato plants of TYLCV infection and heat stress by studying the dominant transcription factor HSFA2 and its interaction with viral proteins. Particular attention was paid to the role of protein aggregation in the processes of tomato adaptation to heat and viral stresses. TYLCV-S and R tomatoes grown at different temperature regimes and the plant responses to combination of HS and virus infection were analyzed.

## Materials and Methods

### Viruses, insects and plants

TYLCV from Israel^24^ and from Jordan^25^ were used in location to inoculate tomato plants (these two isolates are stains from the same virus since their sequences show more than 95% identity). Whiteflies (*Bemisia tabaci* B biotype, termed also MEAM1) were used to inoculate tomato plants. Plantlets (3 weeks after sowing) were caged with adult viruliferous whiteflies for 48 h (approximately 50 whiteflies per plant) as described^26^. Plants from lines 967 and GF967 were used in the greenhouses in Jordan. The TYLCV-susceptible line 967 was obtained from the Jordanian Ministry of Agriculture^27^. The TYLCV-resistant breeding line GF967 was developed in Jordan; it resulted from a cross between line 967 and a TYLCV-resistant line previously bred in Guatemala^28^ based on the resistant germplasm selected in Israel^29^, which was used in our previous studies^7^,^20^,^21^,^22^.

### Heat treatment

Plants were subjected to two types of treatment. 1) In *in vitro* treatments, detached leaves from tomato infected with TYLCV and uninfected, were incubated in dishes at 42–43 ^o^C for 2 h and at 23–25 ^o^C as control. When mentioned, the leaves were let to recover at 23–25 ^o^C for 2 h. 2) Tomato plants of the lines 967 and GF967, infected and uninfected, were divided into two groups maintained in insect-proof greenhouses in Jordan. Plants in the first group were maintained at temperature regime 1 (22–25 ^o^C/18–20 ^o^C, day/night), while plants in the second group were subjected to heat stress at temperature regime 2 (40–45 ^o^C/20–25 ^o^C, day/night). Samples from the upper fully developed leaf were collected from all plants at 0, 7, 14, 21 and 28 days post inoculation (samples of uninfected plants were collected the same day) and immediately stored at −80 ^o^C.

### Extraction of proteins from tomato leaves for SDS-PAGE and sucrose gradient analyses

For SDS polyacrylamide gel electrophoresis (SDS-PAGE) analyses, proteins were prepared as follows. Leaves (about 50 mg pooled from three plants) were minced, frozen in liquid nitrogen and drill-homogenized in a standard SDS-PAGE loading buffer supplemented with 2% SDS. Samples were boiled for 10 min and centrifuged for 40 min at 10,000 × g; about 30 μg protein from the supernatants were subjected to SDS-PAGE.

Native total proteins for sucrose gradient analyses were extracted as follows. Leaves (about 500 mg pooled from three plants) were drill-homogenized in 50 mM Tris–HCl pH 7.5, 80 mM KCl, 10 mM MgCl_2_, 0.2 mM EDTA, 0.5% Nonidet P40, 1 mM dithiothreitol and Complete Protease Inhibitor Mixture (Roche, Mannheim, Germany). Homogenates were incubated on ice for 45 min, vortexed and centrifuged at 1,200 × g for 10 min at 4 ^o^C. The supernatant (0.5 ml) was loaded on top of a 10 ml linear 10–50% sucrose gradient. After centrifugation at 104,000 *g* for 20 h at 4 ^o^C (Beckman SW27 rotor), the content of each gradient tube was divided into 10 fractions as previously described^7^. Aliquots of 50 μl were subjected to 12% SDS PAGE and Western blot analyzed. Each gradient immunodetection was repeated at least five times. Cytoplasmic and nuclear proteins were prepared as previously described^7^.

### *In vitro* immunodetection of viral and plant proteins

Antibodies were prepared against TYLCV CP (Hadar Biotech, Rehovot, Israel) expressed in *E. coli*^7^. Antibodies against HSP70, HSP90, HSP100/ClpB, BiP, Histone H3 and cytoplasmic HSP70 were purchased from Agrisera (Sweden). Anti-HSFA2 was a gift from Prof. K.D. Scharf (Goethe University Frankfurt am Main, Germany). Anti-OE33, a 33-kDa subunit of photosystem II oxygen-evolving complex, was a gift from Prof. Z. Adam (The Hebrew University of Jerusalem, Israel). Incubation with primary antibodies was followed by exposure to secondary goat peroxidase coupled antibodies (Agrisera, Sweden) and by ECL detection (Amersham, UK). Each immunodetection was repeated at least three times for each set of plants (pooled tissues from the upper-most fully developed leaves of three plants).

### Visualization *in situ* of TYLCV CP and plant HSFA2 in tomato leaves

For histological analyses, cross sections of tomato leaves (cut into 0.5 cm squares) were processed as described^7^,^8^. Histological samples were incubated for 12 h at 4 ^o^C with anti-HSFA2 diluted 1:1000 in 2% BSA/MTSB, followed by exposure to Cy3-conjugated anti-rabbit secondary antibody (Jackson Immunoresearch, USA) diluted 1:200. After HSFA2 visualization, slides were incubated again with 2% BSA/MTSB for 2 h and exposed to anti-TYLCV-CP diluted 1:500, followed by Cy3 antibodies (Jackson Immunoresearch, USA) labeling. The samples were inspected using a stereoscopic fluorescence zoom microscope (SMZ1500, Nikon, Japan) and fluorescence microscope (Eclipse 80i, Nikon, Japan); CP was detected as a green fluorescent signal, HSFA2 was detected as a red fluorescent signal. Plant nuclei were stained with DAPI (Thermo Scientific, USA), at 1 μg/ml for 20 min at 25 **°**C, and detected as a blue fluorescent signal.

### Purification of TYLCV proteins expressed in *Escherichia coli* and *in vitro* protein-protein interaction assay

The six full-length TYLCV open reading frames were PCR-amplified using specific primers containing an additional *Nde*I (5′ end) and *Bam*HI (3′ end) restriction site. The amplicons were cloned into the *Nde*I and *Bam*HI sites of plasmid pET-14B (Novagen, USA). The recombinant plasmids were used to transform BL21 *E. coli* cells. Following IPTG induction, the His-tagged TYLCV proteins were purified from recombinant bacteria by Ni-affinity chromatography in the presence of 6 M urea using HisPur Ni-NTA Resin (Thermo Scientific, USA). *E. coli* protein extracts were mixed with equal volume of equilibration buffer (PBS with 20 mM imidazole),incubated for 1 h at RT, washed with two resin-bed volumes of wash buffer (PBS with 25 mM imidazole) and eluted with elution buffer (PBS with 250 mM imidazole). The purified proteins were dialyzed for 16 h at 4 ^o^C against 50 mM Tris-HCl pH 8.6, 5 mM EDTA, 500 mM arginine, 500 mM NaCl, 12.5% glycerol, and centrifuged at 12,000 *g* for 20 min. The TYLCV protein-containing supernatants were stored at −80 ^o^C in 12.5% glycerol and consequently used for protein-protein interaction assays.

For *in vitro* interaction assay, TYLCV His-tagged proteins were diluted four times with equilibration buffer and re-applied to Ni-NTA resin. After 3 h incubation at 4 ^o^C, the resin was washed with wash buffer. About 200 μg of total proteins, extracted from TYLCV-infected and HS-treated tomatoes were loaded on the column for a 12 h incubation at 4 ^o^C. After washing with equilibration buffer and wash buffer, bound proteins were eluted with the elution buffer (this fraction was named “elute 1”), mixed with SDS-PAGE sample buffer and boiled. The remaining proteins were extracted by boiling the resin with sample buffer (“elute 2”). Both kinds of elutes were analyzed by western blotting using anti-HSFA2 antibodies.

### DNA, RNA extractions and qPCR detection of TYLCV DNA

DNA was prepared from 100 mg leaf tissues as described^7^. RNA was prepared from 100 mg leaf tissues using the Tri-Reagent method (Sigma-Aldrich, USA); cDNA was prepared using the EZ-first strand cDNA synthesis kit (Biological Industries, Israel) according to the manufacturer. PCR and reverse transcription PCR (RT-PCR) were conducted in triplicate with three five batches of DNA or RNA. DNA and cDNA were analyzed by qPCR in the presence of SYBR Green I (Takara, Japan) in a Corbett Research Rotor-Gene 6000 cycler. The reaction was as follows: 30 s at 94 ^o^C followed by 40 cycles consisting of 10 s at 94 ^o^C, 30 s at 59 ^o^C, and 20 s at 72 ^o^C. The primers used to amplify a 182 bp fragment of TYLCV (X15656) were sense TCTGTTCAAGGATTTCGTTG and reverse sense GCTGTCGAAGTTCAG CCTTC. As an internal reference for DNA detection, a 183 bp fragment of the tomato “Expressed” housekeeping gene (SGN-U346908) was amplified using the sense CTAAGAACGCTGGACCTAATG and reverse sense TGGGTGTGCCTTTCTGAA TG primers. The expression of the following tomato genes was analyzed using amplicons obtained with the sense and reverse sense primer pairs (5′ to 3′): 180 bp of *β-actin* (TC178617) GGAAAAGCTTGCCTATGTGG/CCTGCAGCTTCCATACC AAT, 66 bp of *HsfA2* (CAA47870) ACCTTGTGGATCAGCTTGGTTTCC/AATAGTGGAGGAGGCCAGAGGAAC, 74 bp of *HsfB1* (CAA39034) GGTGCAGG CGAAGAAACAATGC/TCATATCGGGTGCAACCTTCACG, 77 bp of *Hsp17-C1* (AJ225046) ACTTGGCATCGTGTGGAACG/TGATCCATCTTT GCGTTCTCTGG, 77 bp of *Hsp90-1* (SGN-U312354) TGCGTTCTTGTATGGAAGTCTGC/TGGAC CACTTAGTCACGACCAATC, 71 bp of *Apx1* (DQ099420) ACGATGATATT GTGACACTCTTCCA/AAGCGATGAAACCACAAAAACA, and 190 bp of *Apx2* (DQ099421) TGGGAGGGTGGTGACATATTTT/TTGAAGTGCATAACTTCCCA TCTTT.

## Results

### Tomato plants subjected to elevated temperatures contain increased TYLCV amounts, whether susceptible or resistant to the virus

We have used two tomato lines to analyze the interplay between virus (TYLCV) and heat stress (HS). Line 967 is highly susceptible to TYLCV; line GF967 is resistant to TYLCV. GF967 originated from line 906-7 previously used to study the molecular basis of TYLCV resistance^21^,^28^. Upon infection, 967 presents typical disease symptoms of leaf curling and stunting and contains high levels of virus, while GF967 remains symptomless and contains smaller amounts of virus.

**Figure 1:**
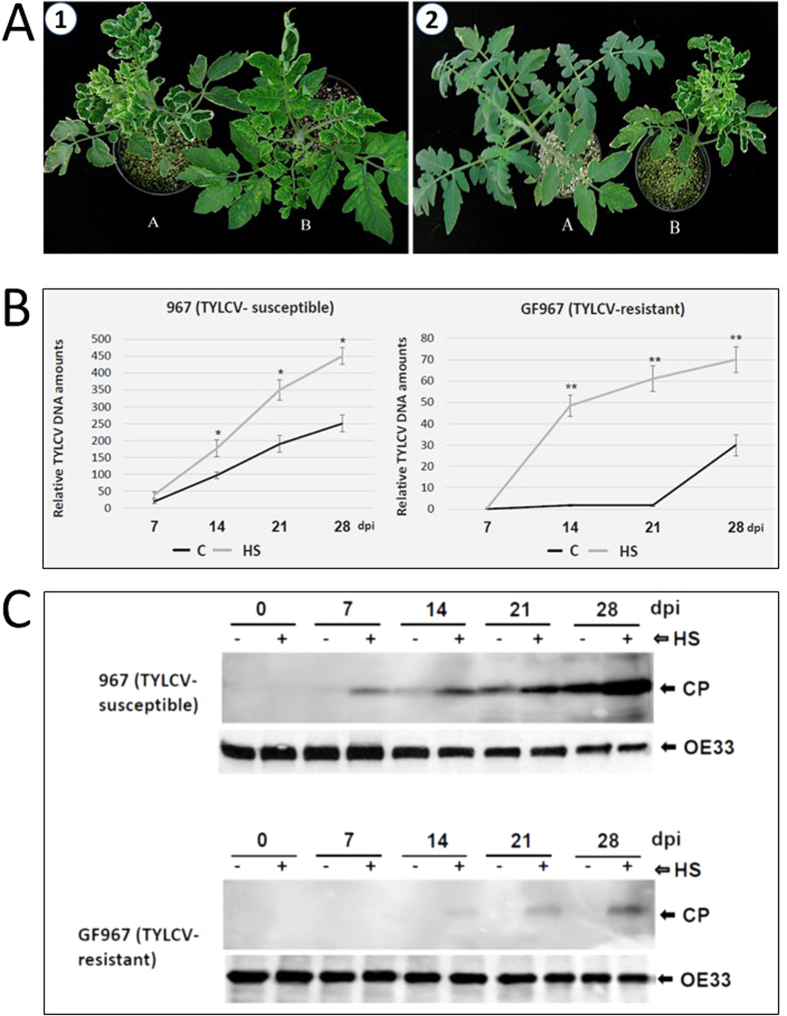
Infected TYLCV-susceptible (967) and resistant (GF967) tomatoes grown at different temperature regimen. (**A**) 1. (**A**) tomato plants of line 967 inoculated with TYLCV and maintained at high temperatures (regime 2: 40–45 ^o^C/20–25 ^o^C, day/night) and (**B**) at normal temperatures (regime 1: 22–25 ^o^C/18–20 ^o^C, day/night). 2: tomato plants of lines GF967 (**A**) and 967 (**B**) inoculated with TYLCV and maintained at high temperatures (40–45 ^o^C/20–25 ^o^C, day/night). Pictures were taken at 28 dpi. (**B)** qPCR estimation of relative TYLCV amounts in untreated (**C**) or heat stress (HS) treated TYLCV-susceptible (line 967) and TYLCV-resistant (line GF967) genotypes. The results were normalized using the tomato *expressed* gene as an internal marker. Results were analyzed using student’s *t* test. One asterisk denotes p < 0.05. Two asterisks denotes p < 0.01. Bars represent the average and standard deviation of the relative expression from five independent biological repeats. (**C**) Western blot analyses of TYLCV CP in tomatoes grown at normal temperatures (−, regime 1) and high temperatures (+, regime 2). Samples were taken every seven days after the onset of viral infection (0, 7, 14, 21, 28 dpi). The OE33 chloroplast protein (OE33) was used as unrelated control.

**Figure 2:**
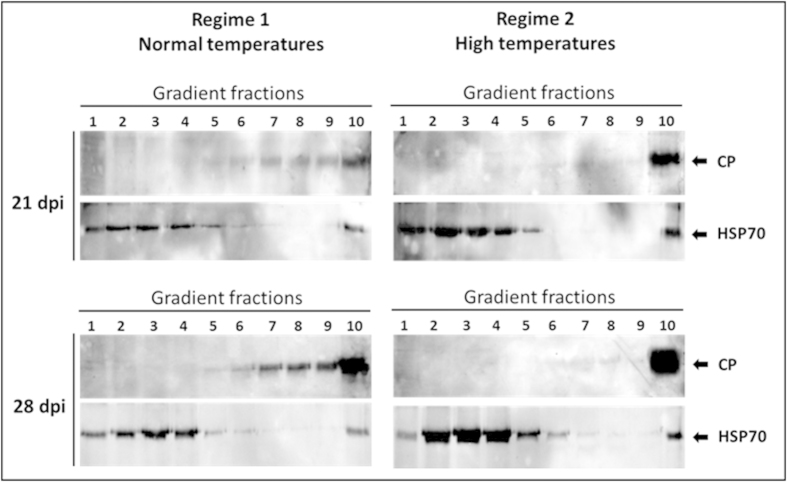
Changes in the patterns of TYLCV CP under constant HS. TYLCV-infected 967 tomatoes were grown at normal (regime, 1: 22–25 ^o^C/18–20 ^o^C, day/night) and at high temperatures (regime 2: 40–45 ^o^C/20–25 ^o^C, day/night). Native proteins, extracted from those tomatoes at 21 and 28 dpi, were subjected to ultracentrifugation on sucrose gradients, which were subsequently divided in 10 fractions; aliquots were analyzed by western blots with antibodies against CP and HSP70 (used as a plant protein marker).

GF967 and 967 tomatoes were grown in two separated greenhouses in the Jordan Valley (Jordan), at two temperature regimen: regime 1 was 22–25 ^o^C/18–20 ^o^C, day/night, regime 2 was 40–45 ^o^C/20–25 ^o^C, day/night. In each greenhouse, two groups of plants were grown, TYLCV–infected and uninfected tomatoes. Growing infected virus-susceptible 967 plants at high temperature exacerbated the magnitude of the disease symptoms already observed at normal temperatures, as shown 28 days after infection (dpi) (Fig. 1A,1). On the contrary, virus-resistant GF967 plants infected the same day as the 967 plants and subsequently grown at high temperature did not show significant disease symptoms (Figs 1A and 2). Samples of young leaves, which developed during the course of TYLCV infection, were collected every 7 days for 28 days. In parallel, samples of uninfected tomatoes were collected the same days. DNA, RNA and proteins were extracted from the leaf samples.

When the virus-susceptible plants (967) were grown at normal temperatures (regime 1), the amounts of viral DNA in leaves (estimated by qPCR) continuously increased during infection. However, when these plants were grown at high temperature (regime 2), the amount of viral DNA accumulating during infection nearly doubled (Fig. 1B,1). On the other hand, when the virus-resistant plants (GF967) were grown at normal temperature (regime 1), the amounts of virus increased significantly only after the third week post-inoculation, albeit to very low levels. However, when these plants were grown at high temperature (regime 2), significant amounts of viral DNA started to accumulate two weeks earlier, increasing steadily, but not reaching the amounts found in the susceptible plants (Figs 1B and 2).

All along the infection, the time-course of TYLCV coat protein (CP) accumulation, estimated by western blot immuno-detection, paralleled that of the TYLCV DNA (Fig. 1C). In line 967, virus CP was detected already at 14 dpi and its amount steadily increased with the development of the infection, a feature shared with other TYLCV susceptible lines^20^. Long-term HS treatment led to a significant increase in the CP amounts all along the infection (Fig. 1C). By comparison, in leaves of GF967 plants grown at temperature regime 1, CP was barely detectable even at 28 dpi; however, when these plants were subjected to high temperatures, the virus CP was conspicuous already at 14 dpi. These results, together with the estimation of viral DNA amounts, indicated that long term HS caused increased TYLCV accumulation in the virus-susceptible and even in the virus-resistant tomatoes.

### Heat stress induces an increase in size of TYLCV CP aggregates

We have previously shown that, with the progress of TYLCV infection, TYLCV CP aggregated in inclusions of increasing size. The appearance of the large CP aggregates was a feature of susceptibility to TYLCV^7^. Here we have analyzed the degree of CP aggregation in lines 967 and GF967, subjected to normal and to high temperatures.

Native proteins, extracted from leaves of the susceptible tomato line 967 at 21 and 28 dpi, were subjected to ultracentrifugation in sucrose gradients. This method has been successfully applied for the detection of plant virus-induced protein aggregates and their separation according to size^7^,^30^. In samples from tomatoes grown at normal temperatures (regime 1), the viral CP at 21 dpi was detected in small/midsized and large aggregates (fractions 6–10) (Fig. 2). Although CP amounts increased during infection, the CP at 28 dpi was present in the same gradient fractions as at 21 dpi. Tomatoes grown at high temperature (regime 2) showed a different CP pattern: whether at 21 or 28 dpi, CP concentrated almost exclusively in the bottom of the gradient (fraction N10), where large aggregates/inclusion bodies are confined. Hence, exposure of TYLCV infected tomatoes to constant HS caused the development of large aggregates/inclusions.

### Heat stress induces different responses in infected TYLCV susceptible (967) and resistant (GF967) tomatoes

**Figure 3:**
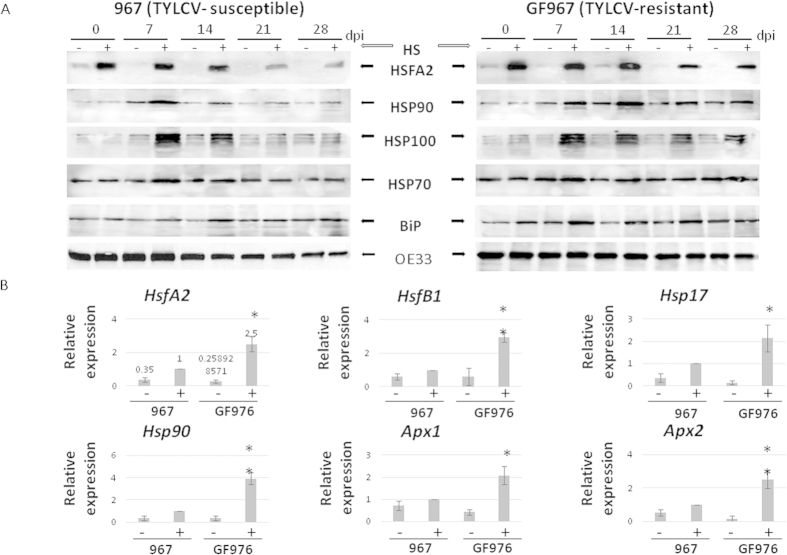
Differences in protein amounts and expression of HS-inducible genes in TYLCV-susceptible (967) and resistant (GF967) tomatoes grown at normal and high temperatures. (**A**) Western blot analyses of plant transcription factor HSFA2 and HSPs in tomatoes grown at normal temperature (−, regime 1) and at high temperatures (+, regime 2). The OE33 chloroplast protein was used as protein loading control. (**B**) qRT-PCR analyses of *Hsf*, *Hsp* and *Apx* gene transcripts of the two lines grown for 21 days at normal temperature (−, regime 1) and at high temperatures (+, regime 2) at 14 dpi. The expression level of each gene was calculated in relation to leaves of susceptible line grown at high temperature. The results were normalized using the *β-actin* gene as an internal marker. Results were analyzed using student’s *t* test. One asterisk denotes p < 0.05. Two asterisks denotes p < 0.01. Bars represent the average and standard deviation of the relative expression from five independent biological repeats; pooled leaves of three different plants were taken for each sample.

In parallel to CP accumulation, we examined the patterns of several HS response proteins in infected 967 and GF967 tomatoes. Compared to plants grown at normal temperatures, whether susceptible or resistant to the virus, the application of constant heat during infection usually caused an increase in the abundances of HSFA2, HSP90, HSP100/ClpB, HSP70 and BiP (an ER HSP70 resident) (Fig. 3A). This heat-induced increase in the amounts of HS-dependent proteins was also observed in non-infected 967 and GF967 tomatoes (Supplementary Fig. 1).

The patterns of HSFA2, HSP90, HSP100/ClpB (but not of HSP70 and BiP) were different in infected 967 and GF967 tomato lines. The activation of HSFA2, HSP90, HSP100/ClpB was less pronounced in line 967, which contained large amounts of virus, than in line GF967 (Fig. 3A). However, even in GF967 plants, HS-induced protein expression was diminished at the late stages of infection, when virus started to be detectable. Hence, there is correlation between TYLCV accumulation and a decline of plant HS response efficiency.

In parallel to the HS-induced proteins, the amounts of *HsfA2*, *HsfB1*, *Hsp17*, *Apx1, Apx2*, *Hsp90* transcript were estimated two weeks after the onset of TYLCV infection (14 dpi) in the leaves of 967 and GF967 tomatoes grown at normal and at high temperatures. The results showed that heat enhanced the accumulation of *HsfA2*, *HsfB1*, *Hsp17*, *Apx1, Apx2*, *Hsp90* transcripts in both lines (Fig. 3B). However in line GF967, the increase was higher than in the 967 line. For example, HS induced a 2.5 fold increase of *HsfA2* transcript amounts in 967 tomatoes compared to a 5 fold increase in GF967; *Hsp17* - was increased 2.5 fold in 967 and 10 fold in GF967. The expression of *Apx* genes was increased only in 0.5–2 fold in 967 and 5–15 fold in GF967. Such differences might be caused by the virus ability to down-regulate the plant HS response, which prevailed in high-virus containing susceptible 967 plants.

### TYLCV accumulation correlates with changed efficiency of HS response

To better understand how TYLCV influences the host plant HS response, we established assays in the laboratory where TYLCV susceptible tomatoes were subjected to HS before and after TYLCV infection. A recovery step was added, where plant samples were returned from high to room temperature (23–25 ^o^C).

**Figure 4:**
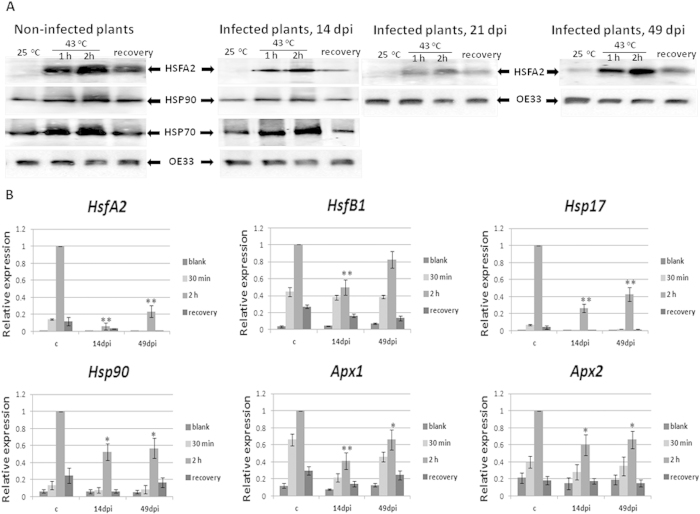
Analyses of proteins and expression profiles of HS-depended genes upon TYLCV infection. Detached leaves from uninfected and TYLCV-infected (14, 21 and 49 dpi) tomato plants of line 967 were incubated at ambient (23–25 ^o^C) and high (42–43 ^o^C) temperatures for 2 h followed by 2 h recovery at ambient temperature. (**A**) Immunodetection of HSFA2 and HSP90/HSP70 in leaf samples. The OE33 chloroplast protein (OE33) was used as unrelated control. (**B**) qPCR transcription profile in uninfected (**c**) and infected (14 and 49 dpi) leaf samples. The expression level of each gene was calculated in relation to uninfected leaves after 2 h heat shock. The results were normalized using the *β-actin* gene as an internal marker. Results were analyzed using analysis of variance (ANOVA). One asterisk denotes p < 0.05. Two asterisks denotes p < 0.01. Bars represent the average and standard deviation of the relative expression from five independent biological repeats. Bars represent the average and standard deviation of the relative expression from five independent biological repeats. Pooled leaves of three different plants were taken for each sample.

Tomato leaf samples were incubated in Petri dishes with water and subjected to a HS (43 ^o^C for 30 min, 1or 2 h) and to a recovery period when mentioned (HS followed by incubation at 23–25 ^o^C for 2 h). Leaf samples incubated with water at room temperature (23–25 ^o^C) were used as controls. HS treatment induced an increase of the amounts of HSFA2, HSP90 and HSP70 proteins in control samples (Fig. 4A). When TYLCV-infected leaves were heat treated, the increase in the amounts of HSFA2 and HSP90 was less pronounced. Conversely, the behavior of HSP70 upon heat shock and recovery was similar in infected and in non-infected plants. The stage of viral infection, but not virus amounts, influenced HS- protein expressions. The efficiency of down-regulation of plant HS response was strong at the beginning of TYLCV infection (14 dpi), reached maximal levels at 21–28 dpi (shown for 21 dpi) and declined at 49 dpi (Fig. 4A). These results corroborated the pattern of *HsfA2* transcription, which was more downregulated at the beginning than at the end of viral infection (14 dpi vs. 49 dpi) (Fig. 4B), even though the viral amounts were maximal levels at the late infectious stages^14^. The transcription levels of the other HS transcription factor *HsfB1* and HSF regulated genes, such as *Hsp17*, *Apx1, Apx2*, *Hsp90*, were at the lowest levels at 14 dpi, while some up-regulation could be observed at 49 dpi (Fig. 4B).

Special interest aroused during the recovery period, when tomato leaf samples were returned to room temperature (23–25 ^o^C) after heat shock. *HsfA2*, *HsfB1*, *Hsp17*, *Apx1, Apx2*, *Hsp90* genes were still less expressed in leaves of TYLCV-infected plants than in uninfected tomatoes (Fig. 4B). The decreased amounts of HSFA2 coincided with a significant reduction in the expression of several heat-inducible genes, as demonstrated for *Hsp17* and *Apx1*/*2* (Fig. 3A). During the recovery period, infected tomatoes did not show the same levels of stress gene expression as those observed in uninfected plants (Fig. 4).

### TYLCV infection changes the intracellular distribution of HSFA2

HSFs exist as inactive proteins mostly found in the cytoplasm. HS causes HSFA2 activation with translocation to the nucleus, where it binds to its target sequences (HSEs) present in the promoter regions of HS genes (30, and references therein).

**Figure 5:**
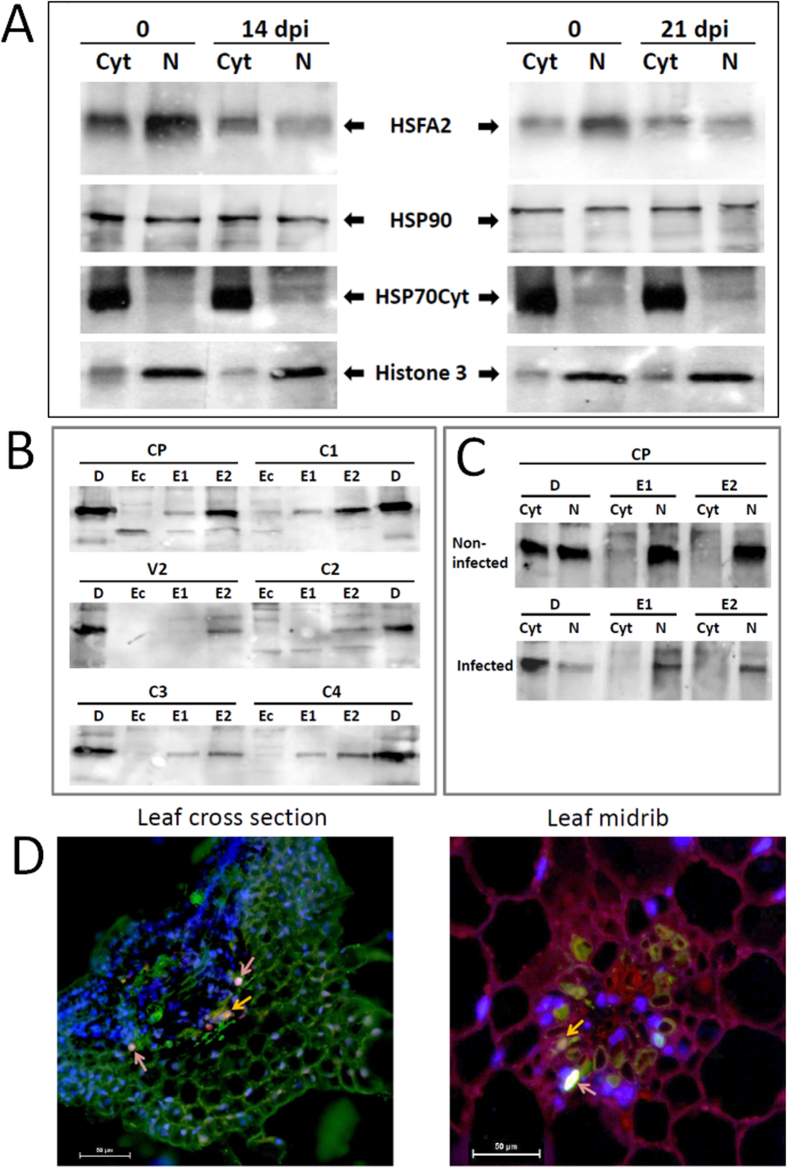
HSFA2 changes in TYLCV-infected tomatoes. (**A**) Immunodetection of HSFA2 in cytoplasmic (Cyt) and nuclear (N) protein extracts of heat shock-treated (1 h) tomato leaves of uninfected vs. infected (14 and 21 dpi) 967 tomatoes. Cytoplasmic HSP70 and nuclear Histone 3 were used as internal markers to assess the purity of the cellular fractions. (**B**) Pull-down assay with purified TYLCV proteins (CP, V2, C1, C2, C3, C4) used as baits for binding with HSFA2 present in leaf total protein extracts. HSFA2 immunodetection revealed potential complexes in plant elutes 1 and/or 2 (E1, E2), but not in *E. coli* protein extracts (Ec). The immunodetection in crude extracts was used as direct control (**D**). (**C**) Immunodetection of HSFA2 in pull down assays, where TYLCV CP preferentially binds HSFA2 in the nuclear protein fraction of uninfected and infected leaves. (**D**) Co-localization of CP and HSFA2 in cytoplasm and nuclei of infected (14 dpi) tomato leaf (cross leaf section and leaf midrib). Fluorescent microscopy using primary anti-CP antisera and Cy2-labeled secondary antibody, primary anti-HSFA2 antisera and Cy3-labeled secondary antibody; nuclei were DAPI stained. Viral CP appears as green, cellular HSFA2 as red, nuclei as blue; CP co-localizing with HSFA2 as yellow (yellow arrow), CP co-localizing with HSFA2 in nuclei as pink (pink arrow). Bar: 50 μm.

HSFA2 nuclear translocation was compared in tomato cells of uninfected vs. infected 967 plants (14, 21 dpi). Detached leaves were exposed to heat shock for 1 h, then protein extracts were separated into cytoplasmic and nuclear fractions, where HSFA2 was immuno-detected (Fig. 5A). The heat shock caused the re-localization of the transcription factor from cytoplasm to nucleus in uninfected tomato cells. In the infected leaves, HSFA2 abundance in the nucleus did not exceed that in the cytoplasm; sometimes HSFA2 levels were even less pronounced in the nucleus (Fig. 5A). Hence, in the presence of TYLCV, a decreased translocation of HSFA2 into the nucleus was observed.

### HSFA2 is able to form complexes with TYLCV proteins

Interaction between HSFA2 in tomato extracts and purified TYLCV proteins was studied by using purified His-tagged viral proteins over-expressed in *E. coli* and bound to His-Bind Resin. Plant total proteins, extracted from infected tomatoes (14 dpi) and heat shock-treated for 1 h, were passed through a Ni-column with bound viral His-tagged CP, V2, C1, C2, C3, and C4. Following elution, the two kinds of bound proteins (described in Materials and Methods) were immuno-detected with anti-HSFA2 antibodies (Fig. 5B). Complexes between HSFA2 and all the six TYLCV proteins were detected. *E. coli* extracts, passed through the same resins, were used as negative controls to confirm the absence of any background originating from the protein purification procedure. By comparison, HSP 70 interacted with only three viral proteins (data not shown, except for CP, described in^7^.

To identify HSFA2-TYLCV complexes in the tomato leaf cellular compartments, proteins from separated cytoplasmic and nuclear fractions were passed through Ni resin columns bound to the viral proteins. Pull down of plant HSFA2 with viral CP was demonstrated in nuclear, but not cytoplasmic protein extracts (Fig. 5C). HSFA2-CP binding was not found in *E. coli* protein extracts (not shown). These results indicated that TYLCV CP was able to bind cellular HSFA2, restricting the capacity of free transcription factor to promote HS-genes transcription in plant nuclei.

The presence of HSFA2-CP complexes was confirmed by *in situ* immuno-fluorescent visualization of plant HSFA2 (red staining) and viral CP (green staining) in leaf sections of symptomatic tomato plants at 21 dpi, heat shock treated *in vitro* 1 h (Fig. 5D). Double *in situ* immuno-detection of HSFA2 and CP demonstrated their co-localized in cytoplasm (yellow staining) and nuclei (pink staining).

### Combined heat and viral stresses induce the aggregation of the cellular chaperones HSP90 and HSP70

As in the case of TYLCV CP (Fig. 2), we investigated whether heat shock alone and heat shock together with TYLCV infection influence the degree of aggregation of two key cellular chaperones, HSP90 and HSP70. After 2 h of heat treatment, tomato leaves were incubated for 2 h at room temperature to recover. We expected that at this time the chaperones returned to their previous soluble state to regulate the protein quality control systems.

**Figure 6:**
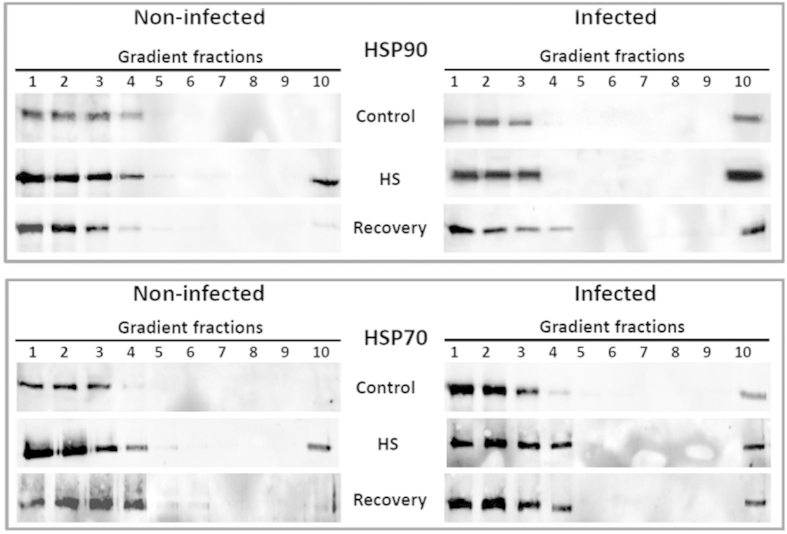
Influence of TYLCV infection on the pattern of HSP90 and HSP70 upon heat shock and recovery. Infected and uninfected tomato leaves were incubated at room temperature (control), and at 42–43 ^o^C for 2 h (heat shock), followed by 2 h recovery at room temperature (recovery). Extracts of native proteins were subjected to ultracentrifugation on sucrose gradients, which were subsequently divided in 10 fractions; aliquots were analyzed by western blots with anti-HSP90 and HSP70 antibodies.

Ultracentrifugation through sucrose gradients showed that in extracts of leaves incubated at normal temperatures, most HSP90 was detected in the gradient fractions containing soluble proteins (fractions 1–2) and small protein complexes (fractions 3–4). TYLCV infection induced the aggregation of some of the HSP90, which appeared in fraction 10 of the gradient, where large protein aggregates are present (Fig. 6, upper panel). Similar results have been shown previously for HSP70 ((4) and Fig. 6, lower panel). Two hours of heat shock (42–43 ^o^C) led to increased amounts of HSPs and their appearance in large aggregates. Heat shock of TYLCV-infected leaves caused an increase in the amounts of chaperone present in gradient fraction 10 (Fig. 6). Recovery from heat shock (2 h at 23–25 ^o^C) led to a decrease in the amount of aggregated HSP90/HSP70 in uninfected tomatoes; the pattern resembled that of leaves incubated at room temperature (Fig. 6). By comparison, in TYLCV-infected leaves that recovered from heat shock, some amounts of aggregated HSPs remained in large aggregates (fraction 10) (Fig. 6). It has to be noted that after recovery there is more HSPs in fraction 10 of infected plants than before heat shock. These results showed that TYLCV enhances heat shock-induced protein aggregation. These aggregates contained viral CP together with at least two cellular chaperones. Restoration of initial chaperone patterns during recovery was impaired in viral infected plants.

## Discussion

The combination of extreme temperature and viral infection occurs quite often in the field in the Middle East and in many tropical regions, impairing crop productivity. It is well known that increasing temperatures facilitate pathogen spread in major food crops^31^. Temperature was identified as the dominant abiotic factor directly affecting herbivorous insects by changing their development, survival, range and abundance^32^. Moreover, many abiotic stresses were shown to weaken the defense mechanisms of plants and enhance their susceptibility to pathogen infection^33^,^34^. Current climate prediction models point to a gradual increase in ambient temperatures in the near future and to an enhancement in the frequency, length and amplitude of heat waves^35^,^36^. Therefore, there is a need to generate crops with enhanced combined tolerance to heat and pathogens.

In the current study, we have highlighted several mechanisms of interaction between HS and TYLCV infection. The effect of HS in promoting virus accumulation was confirmed by subjecting TYLCV susceptible (line 967) and TYLCV-resistant (line GF967) tomato lines grown in near-field conditions. Plants from line 967 grown at high temperatures showed exacerbated symptoms compared to plants grown at milder temperatures (Fig. 1A). Heat application significantly increased the amounts of viral DNA (Fig. 1B) and CP (Fig. 1C) not only in line 967, but also in line GF967. In GF967 tomatoes grown at normal temperatures (regime 1), TYLCV CP was undetectable during prolonged infection (28 dpi). High temperatures (regime 2) led to an increase in CP amounts. Our previous studies indicated that during the course of infection, increasing amounts of TYLCV DNA and CP were present in aggregates of increasing sizes. Moreover, the size expansion of such aggregates was considered as a marker of a successful infection^7^. Heat shock treatment of TYLCV-infected tomato leaves led to the accumulation of viral CP exclusively in large aggregates (Fig. 2).

Comparing the HS-dependent activation profiles of the main heat response players such as HSFs and HSPs in TYLCV-infected and uninfected tomatoes, provides some clues on how tomato plants act under TYLCV and HS. It has been shown that in tomato cell cultures subjected to HS, the constitutively expressed HS transcription factor HSFA1 is complemented by two HS-inducible forms, HSFA2 and HSFB1. Because of its stability, HSFA2 accumulates to high levels in the course of prolonged HS and recovery regimes^37^. Since HSFA2 is an abundant transcription factor with a high activation potential, it is the dominant HSF in tomato upon HS treatment^38^. HSFB1 was suggested to act as a coactivator of HSFA1 during HS^39^,^40^. HSFs may act as molecular sensors to detect the presence of ROS such as H_2_O_2_ and activate downstream stress-responsive genes such as *Apx1/2*, *Hsp17* and others, allowing plants to respond to different environmental conditions^41^. In rice, the induction patterns of HSPs and HSFs following stress treatment such as drought, heat, cold, and salt showed some overlap, but also a specific response to each condition^13^ One of the important results of the current study is the discovery that TYLCV infection is accompanied by a decreased activation of cellular HS response. The transcriptional and/or protein patterns of *HsfA2*/HSFA2, *HsfB1*, *Hsp90*/HSP90 (but not of HSP70 and BiP), HSP100, *Hsp17*, *Apx1* and *Apx2* were significantly decreased in TYLCV-infected tomatoes (Figs 3 and 4). The failure of TYLCV to induce the expression of various plant HSPs has been described previously^20^,^22^. Here we have shown that TYLCV infection inactivates cellular HS response, by decreasing the expression levels of HSFs, HSPs and HSF-regulated proteins (Figs 3 and 4). The current experiments do not completely exclude the possibility that the observed effect on HS response suppression is a secondary effect of changing cell metabolism in the aging infected cells. However, potential TYLCV capacity to suppress the HS response depended on the stage of infection, not on aging of the infected tomato plants. It was minor at 7 dpi (data not shown), started to be significant at 14 dpi and was maximal at 21 dpi, while it has decreased at 49 dpi (Fig. 4), even though the amounts of TYLCV were higher at the later stage of infection. In uninfected tomatoes, some changes in the patterns of HS-inducible proteins during aging were observed (Supplementary Fig. 1), but were much less pronounced than in TYLCV infected plants.

TYLCV infection was accompanied by changes in the intracellular distribution of HSFA2. The decrease in the abundance of HSFA2 occurred mostly in nuclei (Fig. 5A). All the six TYLCV purified proteins could bind to HSFA2 in total protein extracts in *in vitro* assays (Fig. 5B); in addition, CP was shown to be able to interact with HSFA2 in nuclear extracts (Fig. 5C,D). The changes in the HSFA2 patterns point to the reduced presence of free, active nuclear HSFA2, which may be a reason of down-regulated transcriptional levels of HS-genes described through this study (Figs 3 and 4).

In uninfected tomatoes, during the recovery periods that followed heat shock, the key cellular chaperones HSP70 and HSP90 reverted to fractions containing soluble active proteins or protein complexes. In TYLCV-infected plants, this restoration was not complete (Fig. 6). The mobilization of chaperones in large aggregates could weaken the HS response. They could be used by the virus as elements of the protein quality control machinery to reactivate cellular proteins needed for folding and assembly of multimeric protein complexes required for virus replication, transcription and encapsidation. The finding that HSP70 (4), HSP90 (current study), and components of 26S proteasome degradation (6) were recruited into large CP aggregates/inclusion bodies support this hypothesis.

In TYLCV infected tomatoes, a significant reduction in the levels of transcription and translation of the heat-inducible genes can lead to a decreased temperature tolerance, HS response and consequently to reduced cell death. The importance of CD suppression by TYLCV infection was recently described (5). Likely, reduced stress responses supply enough time for successful viral replication. On the other hand, infected tomatoes become much more susceptible to various environmental stresses. HSF signaling is one of the main contributors to resistance against main abiotic stresses and is essential for the maintenance of normal growth and productivity under stress conditions^41^. Stable HSFA2 is expected to confer a stress resistance phenotype while maintaining yield productivity. Indeed, the maintenance of HSFA2 dependent activation of stress response in TYLCV-infected GF967 plants (compared with TYLCV-infected 976 plants) may indicate a better capacity to manage HS and possibly other abiotic stresses. The development of stable multiple stress tolerance traits in important crop plants will improve yields particularly in areas with adverse environmental conditions, and contributing to global food security. One of the successful example of such multiple stress tolerant plants is the TYLCV-resistant GF967 tomato line, which survives under heat and TYLCV stresses. It might be possible to increase the heat tolerance of TYLCV-susceptible plants by pre-inoculating (by agroinfection) seedlings with a TYLCV symptomless mutant lacking 20 amino acids near the N-terminus of the CP^42^, and therefore not transmissible by whiteflies, before planting in the field. We expect that the mutant will have the same capacity to suppress the HS response and to increase heat tolerance as the wild type virus, though this has to be proven experimentally.

## Additional Information

**How to cite this article**: Ghandi, A. *et al.* *Tomato yellow leaf curl virus* infection mitigates the heat stress response of plants grown at high temperatures. *Sci. Rep.* **6**, 19715; doi: 10.1038/srep19715 (2016).

## Supplementary Material

Supplementary Information
