# Corrigendum: *Tomato yellow leaf curl virus* infection mitigates the heat stress response of plants grown at high temperatures

**DOI:** 10.1038/srep25284

**Published:** 2016-05-11

**Authors:** Ghandi Anfoka, Adi Moshe, Lilia Fridman, Linoy Amrani, Or Rotem, Mikhail Kolot, Mouhammad Zeidan, Henryk Czosnek, Rena Gorovits

Scientific Reports 6: Article number: 19715;10.1038/srep19715 Published online: 01212016; Updated: 05112016

The original version of this Article contained errors in the spelling of the authors Ghandi Anfoka, Adi Moshe, Lilia Fridman, Linoy Amrani, Or Rotem, Mikhail Kolot, Mouhammad Zeidan, Henryk Czosnek and Rena Gorovits which were incorrectly given as Anfoka Ghandi, Moshe Adi, Fridman Lilia, Amrani Linoy, Rotem Or, Kolot Mikhail, Zeidan Mouhammad, Czosnek Henryk and Gorovits Rena respectively.

In addition, the ‘How to cite this article’ section quoted an incorrect abbreviation for Ghandi Anfoka.

These errors have now been corrected in the PDF and HTML versions of the Article.

