# Supplementary material for: Tomato yellow leaf curl virus infection mitigates the heat stress response of plants grown at high temperatures
Source: Sci Rep. 2016 Jan 21;6:19715. doi: 10.1038/srep19715 (PMC4726131; doi:10.1038/srep19715)
Supplement: Supplementary Information [file srep19715-s1.pdf]

Supplementary Figure 1

***Tomato yellow leaf curl virus* infection mitigates the heat stress response of plants grown at high temperatures.**

Anfoka G., Moshe A., Fridman L., Amrani L., Rotem O., Kolot M., Zeidan M., Czosnek H., Gorovits R.

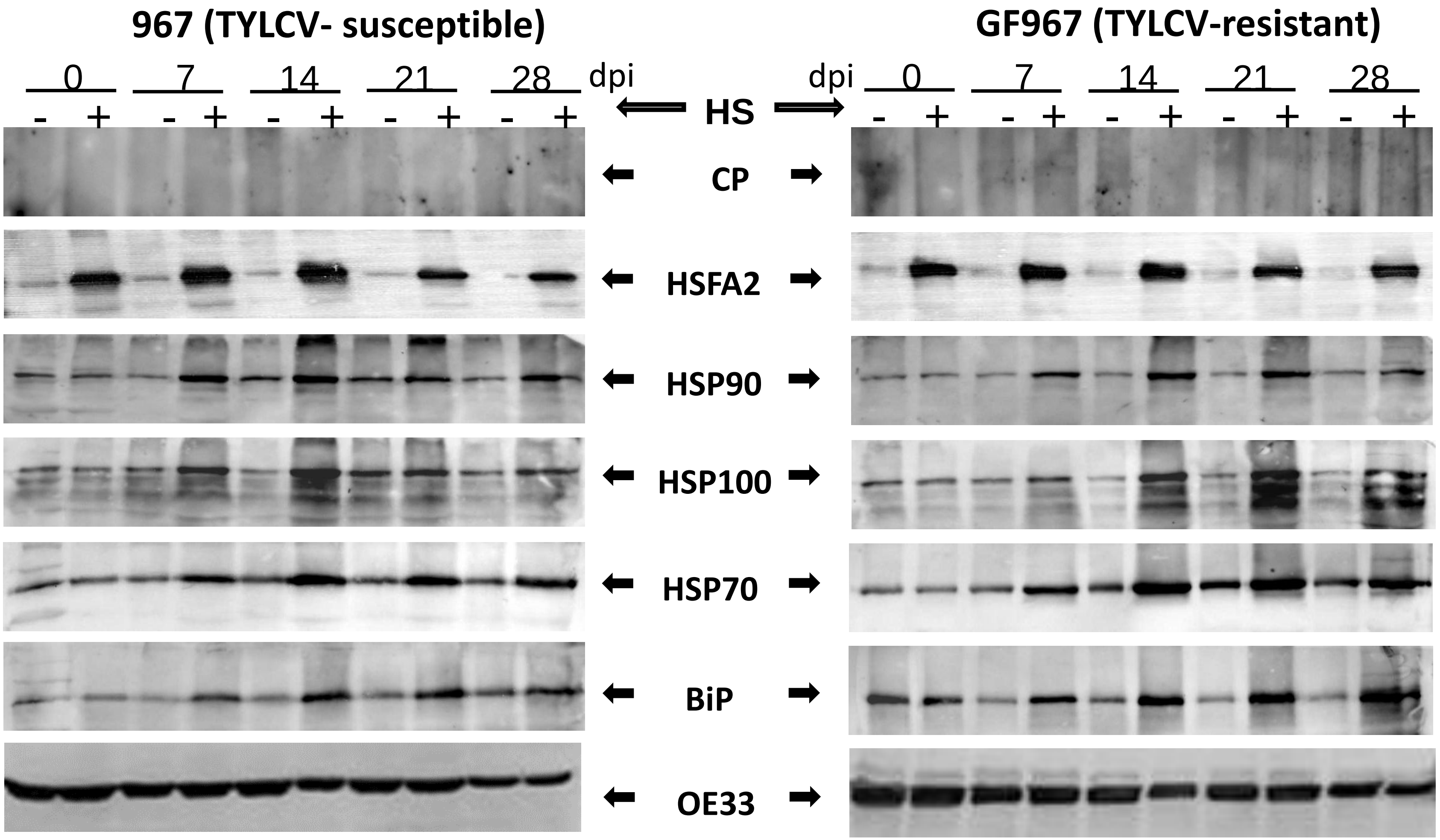

Supplementary Figure 1. Comparative behavior of uninfected 967 and GF967 tomatoes at high (HS) and normal temperatures. Immunodetection of plant transcription factor HSFA2 and HSP90, HSP100, HSP70, BiP in leaves of tomato grown at normal (-, regime 1) and high (+, regime 2) temperatures The OE33 chloroplast protein was used as protein loading control. Compare with Fig. 3A.
